# Long-Term Phytoremediation of Coastal Saline Soil Reveals Plant Species-Specific Patterns of Microbial Community Recruitment

**DOI:** 10.1128/mSystems.00741-19

**Published:** 2020-03-03

**Authors:** Xiaogai Wang, Ruibo Sun, Yinping Tian, Kai Guo, Hongyong Sun, Xiaojing Liu, Haiyan Chu, Binbin Liu

**Affiliations:** a Key Laboratory of Agricultural Water Resources, Hebei Key Laboratory of Soil Ecology, Center for Agricultural Resources Research, Institute of Genetics and Developmental Biology, Chinese Academy of Sciences, Shijiazhuang, China; b University of Chinese Academy of Sciences, Beijing, China; c Institute of Soil Science, Chinese Academy of Sciences, Nanjing, China; Lawrence Berkeley National Laboratory

**Keywords:** costal saline soil, salt-tolerant plant, phytoremediation, root-associated microbiomes

## Abstract

Soil salinization is one of the major land degradation processes that decreases soil fertility and crop production worldwide. In this study, a long-term coastal saline soil remediation experiment was conducted with three salt-tolerant plant species: Lycium chinense Mill. (LCM), Tamarix chinensis Lour. (TCL), and Gossypium hirsutum Linn. (GHL). The three plant species successfully remediated the saline soil but showed different efficacies. The archaeal, bacterial, and fungal communities in barren soil and in four rhizocompartments (distal-rhizosphere soil, proximal-rhizosphere soil, rhizoplane, and endosphere) of the three plant species were assessed. All three plant species significantly decreased the richness of the archaeal communities but increased that of the bacterial and fungal communities in both the rhizosphere and rhizoplane compared with those in the barren soil. The archaeal and bacterial community structures were strongly influenced by the rhizocompartment, while specific fungal communities were recruited by different plant species. The microbial taxa whose abundance either increased or decreased significantly during remediation were identified. Soil electrical conductivity (EC) was identified as the main factor driving the variation in microbial community composition between the remediated and barren soil, and total nitrogen (TN), total carbon (TC), and available potassium (AK) were the main factors driving the differences among plant species. This report provides new insights into the responses of the root zone microbial communities of different salt-tolerant plant species during phytoremediation.

**IMPORTANCE** Despite knowing that phytoremediation by salt-tolerant plants is an effective technology for ameliorating saline soils and that microorganisms contribute significantly to plant stress tolerance and soil fertility, we still lack a comprehensive understanding of how microbes respond to the growth of salt-tolerant plants and the subsequent decline in soil salinity. The results of this study revealed different response patterns among bacterial, archaeal, and fungal communities and indicated that the decline in archaeal abundance might be a sign of successful remediation of coastal saline soils. The recruitment of specific fungal communities by different plant species indicated the importance of fungi in plant species-specific remediation functions. We also identified the taxa that may play key roles during remediation, and these taxa could potentially be used as indicators of phytoremediation. Overall, these findings highlight the importance of microbes in the phytoremediation of saline soil and suggest that the mechanisms involved are plant species specific.

## Abstract (precis)

Despite knowing that phytoremediation by salt-tolerant plants is an effective technology for ameliorating saline soils and that microorganisms contribute significantly to plant stress tolerance and soil fertility, we still lack a comprehensive understanding of how microbes respond to the growth of salt-tolerant plants and the subsequent decline in soil salinity. The results of this study revealed different response patterns among bacterial, archaeal, and fungal communities and indicated that the decline in archaeal abundance might be a sign of successful remediation of coastal saline soils. The recruitment of specific fungal communities by different plant species indicated the importance of fungi in plant species-specific remediation functions. We also identified the taxa that may play key roles during remediation, and these taxa could potentially be used as indicators of phytoremediation. Overall, these findings highlight the importance of microbes in the phytoremediation of saline soil and suggest that the mechanisms involved are plant species specific.

## INTRODUCTION

Soil salinization is a growing global problem that influences plant growth and crop productivity (1–3). Salinity stress negatively affects photosynthesis, respiration, and protein synthesis in plant cells (4, 5). Currently, approximately 1.1 × 10^9^ ha of land worldwide is salt affected, and this number is still increasing by 1.5 million hectares per year (6). In China, there are more than 3.6 × 10^7^ ha of salt-affected lands, including 9.2 × 10^6^ ha of arable lands, which account for 6.62% of the total arable land in the country (7). One of the important areas of salt-affected land is the coastal saline area formed due to seawater intrusion and the dry climate. In China, approximately 10^6^ ha of coastal areas are salt affected (8). These soils usually have high salinity and low nutrient content and therefore substantially restrict plant growth. The remediation of coastal saline soil not only is important for ecological restoration but can also alleviate the lack of arable lands to meet the increasing demand for agricultural production.

Phytoremediation, also known as vegetative bioremediation, is an approach for saline soil remediation through the cultivation of salt-accumulating or salt-tolerant plants and is perceived as a sustainable and cost-effective technique (9–11). The successful growth of salt-tolerant plants in salt-affected areas (10, 12) and the various remediation mechanisms employed by plants (13) have been reported in previous studies. Basically, the two main mechanisms involved are based on either the exclusion of salt by the roots or the control of salt concentration and distribution (14). The plant species used for phytoremediation are mainly halophyte, hyperaccumulator, salt-tolerant, or transgenic plants (12). Tamarix chinensis has been reported to successfully reduce the salt concentration in saline soils and increase the abundance of soil nutrients (15, 16). Lycium chinense is also classified as a halophyte (17) and can grow in highly saline soil (18). Gossypium hirsutum, commonly known as upland cotton, is classified as a salt-tolerant plant, although the levels of salt tolerance differ among cultivars (19, 20). Although using these plants for the purpose of phytoremediation in coastal saline soils has been reported, a comprehensive comparison of remediation efficacies and its underlying mechanisms across plant species, particularly in long-term treatments, has not been performed.

Plant-microbe interactions can greatly influence plant tolerance to salt stress (21). Microbial communities associated with plant roots are crucial for plant growth and health and are therefore referred to as the “second genome” of the plant (22). Previous studies have demonstrated that plant growth-promoting bacteria (PGPB) can increase plant resistance to the adverse effects of salinity (23, 24), and halotolerant bacteria improve plant growth under conditions of saline stress through direct or indirect mechanisms (25). Arbuscular mycorrhizal fungi (AMF) can improve the resistance of plants under conditions of salinity stress by promoting nutrient uptake, water absorption capacity, and osmolyte accumulation (14). Since the microbial communities in the root zone compartments acquire nutrients largely from root exudates and plant litter, salt-tolerant plant species can strongly influence microbial community composition and function (15, 26, 27). This necessitates a plant species-specific investigation of the recruitment of the microbial community in the root zone compartments of salt-tolerant plants.

Previous studies that focused on the remediation of salt-affected soil demonstrated that salt-tolerant plants strongly influence soil physicochemical properties (28–31). However, there is limited knowledge on how different salt-tolerant plant species shape their root zone microbial communities. Investigations of the efficacy of salt-tolerant plants for saline soil remediation and studies linking the remediation efficacy to the host-specific microbial communities are of great importance for the development of phytoremediation techniques.

In this study, three salt-tolerant plant species, Lycium chinense Mill. (LCM), Tamarix chinensis Lour. (TCL), and Gossypium hirsutum Linn. (GHL), were employed in a long-term (∼15-year) field experiment for costal saline soil remediation. All three plant species successfully improved the soil fertility but with different efficacies. The soil properties and microbial (archaeal, bacterial, and fungal) communities in barren soil and in four rhizocompartments (distal-rhizosphere soil, proximal-rhizosphere soil, rhizoplane, and endosphere) were investigated. The aims of the study were (i) to compare the remediation efficacies of the different plant species with respect to soil properties, (ii) to demonstrate the impacts of the plant species on soil microbial communities and potential ecological functions, and (iii) to explore the mechanisms that shape the soil microbial communities across the plant species.

## RESULTS

### Effects of phytoremediation on soil properties.

The physicochemical characteristics of the barren, distal-rhizosphere, and proximal-rhizosphere soils are summarized in Table 1. The majority of soil properties differed between the barren soil and the phytoremediated soils. The barren soil was alkaline, and the soil pH was not significantly changed in the GHL and LCM fields, but the soil in the TCL field was more alkaline than the barren soil. The electrical conductivity (EC) was highest in the barren soil (8,260 ± 2,101 μS cm^−1^) and dramatically declined in the phytoremediated soils. The soil organic carbon (SOC) content in the TCL and LCM treatments was higher than that in the barren soil but not significantly changed in the GHL treatment; the highest SOC value was 1.28 ± 0.38%, which was observed in the LCM distal-rhizosphere soil sample. The NO_3_^−^-N concentration of the barren soil was significantly lower than that of the distal-rhizosphere soils in all three remediation treatments. The concentrations of NH_4_^+^-N in the distal-rhizosphere and proximal-rhizosphere samples in the TCL and LCM were higher than those in the barren soil and the GHL treatment. The highest concentration of NH_4_^+^-N was 7.52 ± 2.23 mg kg^−1^ in the LCM distal-rhizosphere soil sample, and the lowest concentration was 0.10 ± 0.02 mg kg^−1^ in the GHL distal-rhizosphere soil sample. The level of soil-available P (AP) was generally lower in the proximal-rhizosphere samples than in the distal-rhizosphere samples, while the reverse trend was observed for the available K (AK). The highest AK, AP, and total carbon (TC) levels were detected in the LCM. The highest total nitrogen (TN) level and the lowest C/N ratio were observed in the GHL proximal-rhizosphere soil sample.

**TABLE 1:** Basic physicochemical properties of the barren soil and rhizosphere soils under conditions of phytoremediation treatments^*a*^

Property^*b*^	Soil	Treatment			
		Barren land	GHL	LCM	TCL
pH	Distal rhizosphere	8.45 ± 0.13b	8.62 ± 0.26b	8.09 ± 0.42b	9.29 ± 0.20a
	Proximal rhizosphere		8.89 ± 0.15a	8.28 ± 0.01b	8.88 ± 0.11a

EC (μs cm^-1^)	Distal rhizosphere	8,260 ± 2,101a	548 ± 141b	914 ± 198b	440 ± 141b
	Proximal rhizosphere		244 ± 72a	193 ± 63a	289 ± 44a

SOC (%)	Distal rhizosphere	0.55 ± 0.08b	0.56 ± 0.06b	1.28 ± 0.38a	0.87 ± 0.21ab
	Proximal rhizosphere		0.54 ± 0.04b	1.08 ± 0.10a	0.92 ± 0.10a

NH_4_^+^-N (mg kg^-1^)	Distal rhizosphere	0.32 ± 0.08b	0.10 ± 0.02b	7.52 ± 2.23a	2.34 ± 1.79b
	Proximal rhizosphere		0.20 ± 0.06b	4.04 ± 0.32a	3.84 ± 0.10a

NO_3_^−^-N (mg kg^-1^)	Distal rhizosphere	2.70 ± 1.22c	21.61 ± 5.20a	14.50 ± 1.18ab	9.99 ± 3.17b
	Proximal rhizosphere		2.44 ± 1.53a	8.13 ± 0.60b	3.40 ± 0.59a

AK (mg kg^-1^)	Distal rhizosphere	126 ± 9b	129 ± 28b	273 ± 27a	241 ± 49a
	Proximal rhizosphere		174 ± 27b	316 ± 40a	392 ± 30a

AP (mg kg^-1^)	Distal rhizosphere	3.1 ± 0.7b	20.3 ± 6.3b	158.4 ± 53.5a	3.7 ± 1.5b
	Proximal rhizosphere		17.3 ± 9.9a	8.2 ± 1.7a	2.6 ± 0.2a

TC (%)	Distal rhizosphere	1.43 ± 0.13b	1.88 ± 0.04ab	3.57 ± 1.60a	1.89 ± 0.07ab
	Proximal rhizosphere		1.80 ± 0.02b	2.31 ± 0.07a	1.92 ± 0.16b

TN (%)	Distal rhizosphere	0.07 ± 0.01b	0.07 ± 0.01b	0.28 ± 0.12a	0.12 ± 0.02b
	Proximal rhizosphere		1.07 ± 0.63a	0.16 ± 0 0.01b	0.12 ± 0.01b

C:N	Distal rhizosphere	19.2 ± 0.38b	28.2 ± 3.0a	12.3 ± 0.3c	16.1 ± 2.1bc
	Proximal rhizosphere		2.9 ± 2.2b	14.2 ± 1.0a	16.4 ± 0.4a

a Values represent means ± standard deviations (SDs) (*n* = 3). Values within a row followed by different lowercase letters are significantly different (*P *< 0.05, Duncan’s test).

b EC, electrical conductivity; NH_4_^+^-N, ammonia nitrogen; NO_3_^−^-N, nitrate nitrogen; AK, available potassium; AP, available phosphorus; TC, total carbon; TN, total nitrogen; SOC, soil organic carbon.

### Soil microbial communities under different treatment conditions.

**FIG 1:**
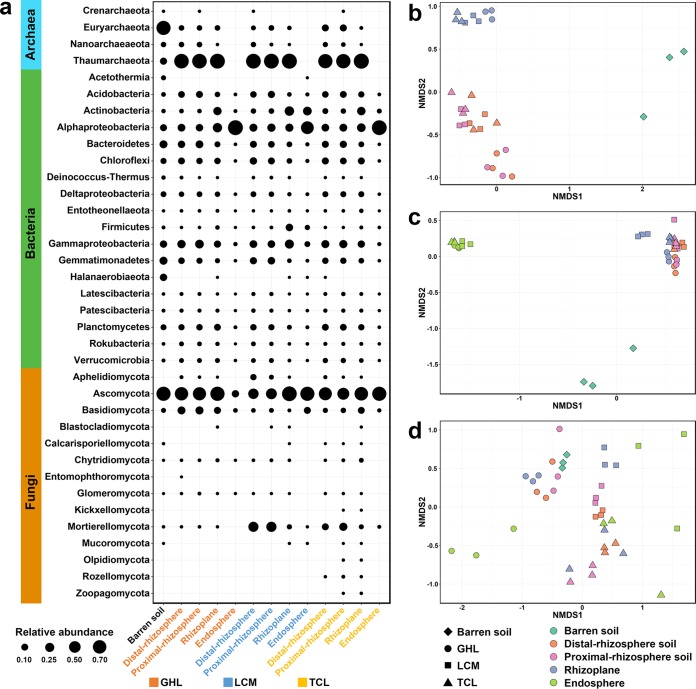
(a) The composition of archaeal, bacterial, and fungal communities in the barren soil and in the four rhizocompartments of the three salt-tolerant plant species. (b to d) NMDS plots of archaeal communities (b), bacterial communities (c), and fungal communities (d) based on Bray-Curtis distances.

In this study, the distal-rhizosphere soil was considered the phytoremediated soil and used for comparison analysis with the barren soil to illustrate phytoremediation performance. In total, 110,118 (archaeal 16S rRNA gene), 949,686 (bacterial 16S rRNA gene), and 1,562,218 (fungal ITS1 region) high-quality reads were obtained from all samples. At the phylum level, the community structure varied between the barren soil and the distal-rhizosphere soils. The archaeal community in barren soil was dominated by *Euryarchaeota* organisms, which were much less abundant in phytoremediated soils (Fig. 1a). *Thaumarchaeota* was the dominant phylum in the rhizoplane and rhizosphere soils of all three remediation treatments, with relative abundance of 86.2% to 99.6%, which was significantly higher than that in barren soil. Archaea were not detected in the endosphere of any investigated salt-tolerant plants (Fig. 1a). In total, 18 dominant phyla (or classes, in the case of *Proteobacteria*) with relative abundances of >1% were identified in the bacterial community (Fig. 1a). The most abundant bacterial phyla were *Proteobacteria*, *Bacteroidetes*, *Actinobacteria*, *Firmicutes*, *Chloroflexi*, *Acidobacteria*, *Planctomycetes*, and *Gemmatimonadetes*, accounting for 75.2% to 99.6% of the bacterial community. *Halanaerobiaeota* were detected only at a high relative abundance (12.7%) in barren soil. Phytoremediation increased the relative abundances of *Acidobacteria*, *Alphaproteobacteria*, and *Gammaproteobacteria*. Moreover, the microbial communities of different rhizocompartments and plant species responded differently to phytoremediation. With respect to bacteria, *Alphaproteobacteria* were enriched within endosphere samples compared with proximal-rhizosphere and distal-rhizosphere soil samples, and *Actinobacteria* were significantly enriched within rhizoplane samples. With respect to fungi, a total of 14 fungal phyla were identified (Fig. 1a). *Ascomycota* was the most dominant phylum, accounting for 36.9% to 93.9% of the fungal community in all the samples except the GHL endosphere sample, in which its relative abundance was 12.2%. *Mortierellomycota* were significantly enriched in distal-rhizosphere and proximal-rhizosphere soil samples under the TCL and LCM treatment conditions. *Olpidiomycota*, *Rozellomycota*, and *Zoopagomycota* were present only in the TCL-remediated soils. We also found that *Basidiomycota* organisms had a higher relative abundance in the rhizosphere and rhizoplane of GHL than the other plant species.

The microbial colonization of roots is a complex and intricate process (32, 33). To illustrate the location preference of the microbial communities in the root zone of different plant species, we identified the microbial communities that were enriched in the rhizoplane and/or endosphere compared with those in the rhizospheres. The operational taxonomic units (OTUs) that were significantly enriched in the rhizoplane and/or endosphere of different salt-tolerant species and had abundances of >1% are shown in Tables S1 and S2 in the supplemental material. We observed that the three salt-tolerant plant species recruited particular bacterial and fungal populations in the rhizoplane and endosphere. For example, we found that *Pantoea* was enriched in the rhizoplane or endosphere of GHL and LCM, and *Streptomyces* and *Purpureocillium* were enriched in TCL and LCM. However, *Ilumatobacter*, *Bacillus*, and *Rhodomicrobium* were enriched only under GHL, LCM, and TCL conditions, respectively (Tables S1 and S2).

10.1128/mSystems.00741-19.2 TABLE S1 Dominant bacterial OTUs enriched in the rhizoplane and/or endosphere. Download Table S1, DOCX file, 0.04 MB. Copyright © 2020 Wang et al. 2020 Wang et al. This content is distributed under the terms of the Creative Commons Attribution 4.0 International license.

10.1128/mSystems.00741-19.3 TABLE S2 Dominant fungal OTUs enriched in the rhizoplane and/or endosphere. Download Table S2, DOCX file, 0.04 MB. Copyright © 2020 Wang et al. 2020 Wang et al. This content is distributed under the terms of the Creative Commons Attribution 4.0 International license.

The variation in the soil microbial communities of the different treatments was further visualized by nonmetric multiple-dimensional scaling (NMDS) ordination based on Bray-Curtis distance (Fig. 1b to d). The two-dimensional plots showed that phytoremediated soils were clearly separated from the barren soil, indicating that salt-tolerant plants greatly changed the soil microbial communities. In addition, the distributions of archaeal, bacterial, and fungal communities in the plots were different. The archaeal and bacterial communities were generally clustered based on the rhizocompartments, except the communities between the distal and proximal rhizosphere were not clearly separated (Fig. 1b and c). In contrast, the fungal communities were clearly separated by plant species (Fig. 1d).

**FIG 2:**
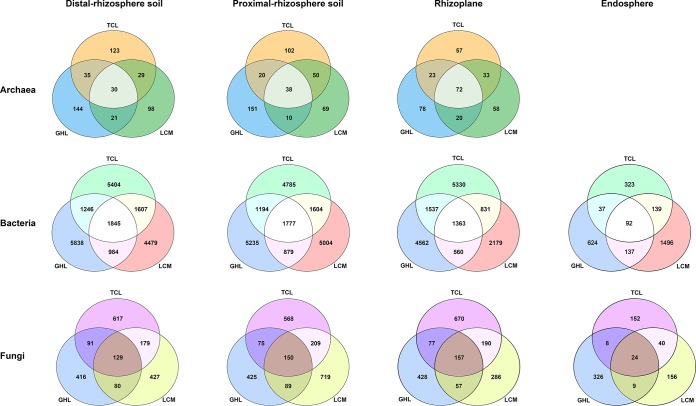
Venn diagrams showing the number of shared and unique OTUs of archaeal, bacterial, and fungal communities in the four rhizocompartments of the three salt-tolerant plant species.

The differences in soil microbial communities among the three salt-tolerant plant species were further investigated through Venn diagrams (Fig. 2). In general, in each rhizocompartment, large proportions of the archaeal, bacterial, and fungal OTUs were detected only in conjunction with a specific plant species, and a small portion of OTUs were common across the three plant species. The number of archaeal OTUs shared among the three plant species was higher in the rhizoplane than in the distal-rhizosphere and proximal-rhizosphere soils. There were more unique bacterial OTUs in the GHL distal rhizosphere and proximal rhizosphere than in these compartments under LCM and TCL conditions. However, TCL had the highest number of unique rhizoplane bacterial OTUs, and LCM had the highest number of unique endosphere bacterial OTUs. LCM had the highest number of unique proximal-rhizosphere fungal OTUs but the lowest number of unique rhizoplane fungal OTUs. TCL had the highest number of unique distal-rhizosphere and rhizoplane fungal OTUs but the lowest number of unique endosphere fungal OTUs, and GHL had the highest number of unique endosphere fungal OTUs but the lowest number of unique proximal-rhizosphere fungal OTUs.

**FIG 3:**
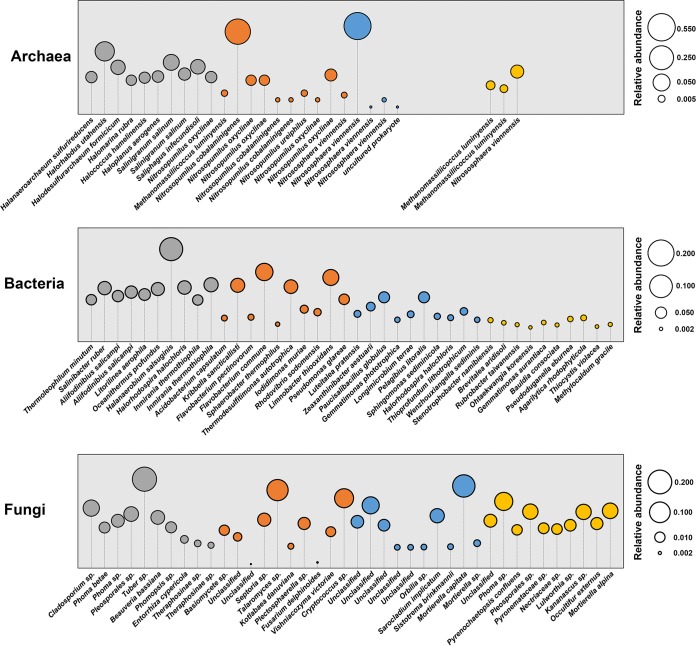
Indicator species (OTUs) for archaeal, bacterial, and fungal communities in barren soil (gray) and the distal rhizosphere of GHL (orange), LCM (blue), and TCL (yellow). Identical taxon names represent different OTUs that have been classified into that taxon.

The term “indicator species” refers to organisms whose presence, absence, or abundance can serve as a measure of environmental conditions (34). In this study, the indicator species were determined to identify the OTUs that were specifically associated with the barren soil and the three phytoremediated soils (represented by the distal-rhizosphere soils) (Fig. 3). The top 10 indicator species (OTUs) in terms of relative abundance in the distal-rhizosphere soils are shown in Fig. 3. The dominant archaeal indicator OTUs in barren soil were most closely related to halophilic taxa, while most of those in phytoremediated soils were annotated as *Nitrosopumilus* or *Nitrososphaera*, as well as some taxa in *Methanomassiliicoccus* in the GHL and TCL treatments. There were also many halophilic taxa within the bacterial indicator OTUs of barren soil, such as Salinibacter ruber, Halanaerobium salsuginis, and Halorhodospira halochloris. Some indicator species in phytoremediated soils have been reported to be capable of degrading complex organic compounds, such as Flavobacterium pectinovorum (35) under GHL conditions and Brevitalea aridisoli (36) under TCL conditions. Some halophilic bacteria under LCM conditions were also identified as indicator OTUs, such as Halorhodospira halochloris (37) and Wenzhouxiangella sediminis (38). Most of the fungal indicators were saprotrophs, but the taxonomy could not be determined by FUNGuild, and there were also some potential animal and plant pathogens, such as Beauveria bassiana in the barren soil and Fusarium delphinoides under GHL conditions.

### Microbial alpha diversity.

**FIG 4:**
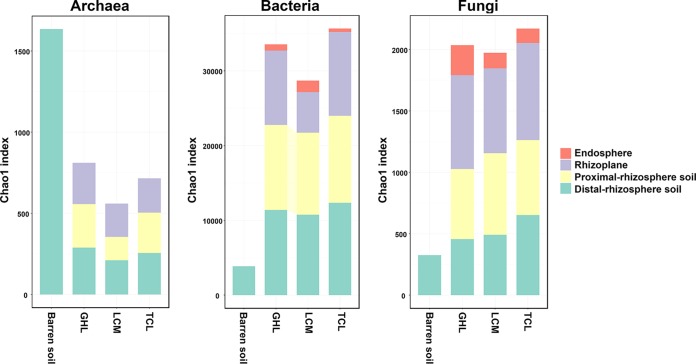
Chao1 richness of archaeal, bacterial, and fungal communities in the barren soil and the four rhizocompartments of the three salt-tolerant plant species.

Chao1 richness was calculated to compare the levels of microbial diversity in different treatments and rhizocompartments (Fig. 4). The archaeal richness was highest in the barren soil and was dramatically lower in the phytoremediated soils. Significantly lower archaeal richness was observed in the proximal rhizosphere of LCM than in those of GHL and TCL, while there were no significant differences in archaeal richness in the rhizoplanes of the three plant species. In contrast, bacterial richness was significantly higher in the three phytoremediated soils than in the barren soil. The bacterial richness showed a decreasing trend from the distal rhizosphere to the endosphere, although the difference between the distal-rhizosphere and proximal-rhizosphere soil samples was not statistically significant. TCL had higher bacterial richness in the rhizoplane and lower richness in the endosphere than was seen with the other two plant species. Similarly to the bacterial communities, the Chao1 richness values of fungal communities of the phytoremediated soils were increased compared to those of the barren soil; TCL had the highest richness in the distal-rhizosphere soil and the rhizoplane, and the GHL and TCL treatments presented the highest and the lowest fungal diversity, respectively, in their endospheres. In contrast to the bacterial community, the fungal alpha diversity showed an increasing trend from the distal rhizosphere to the rhizoplane. As expected, the endophytic microbial communities of all three plant species were less diverse than those of the other root compartments.

### Predictive functional profiling.

**FIG 5:**
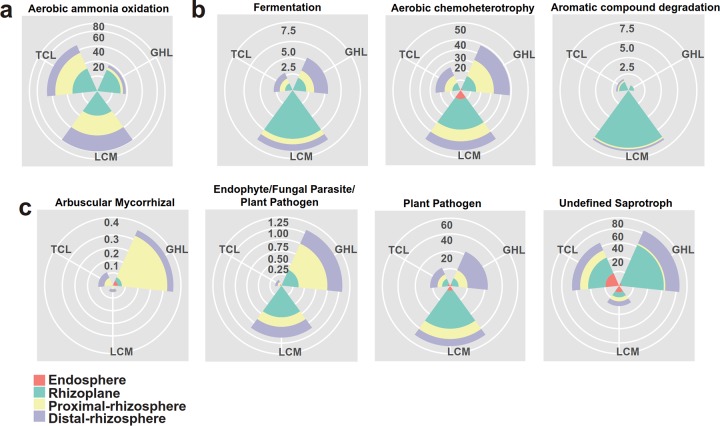
Functional predictions of archaeal (a), bacterial (b), and fungal (c) communities in the four rhizocompartments of the three salt-tolerant plant species.

Ecological functions were predicted by using Functional Annotation of Prokaryotic Taxa (FAPROTAX) for the bacterial and archaeal communities and FUNGuild for the fungal communities in the barren soil and the three phytoremediated soils (Fig. 5; see also Tables S3, S4, and S5). FAPROTAX analysis showed 5 total functional categories in the archaeal communities. The most prominent predicted function was aerobic ammonia oxidization, and the relative abundance of this category was higher in the phytoremediated soils than in the barren soil (Table S3). In addition, the abundance of this functional group showed an increasing trend from the distal rhizosphere to the rhizoplane (Fig. 5a). With respect to the bacterial communities, 60 functional groups were identified from all samples. Fermentation and aerobic chemoheterotrophy were the dominant predicted functions in the distal-rhizosphere, proximal-rhizosphere, and rhizoplane samples, while intracellular parasites were the dominant predicted functional group in the endosphere (Fig. 5b; see also Table S4). FUNGuild analysis revealed profound effects of phytoremediation on the predicted fungal functions. In particular, the dominant functional groups, which were the undefined saprotrophs and the arbuscular mycorrhizal and endophyte/fungal parasite/plant pathogens, exhibited high variability in relative abundance among the rhizocompartments and across the plant species (Fig. 5c; see also Table S5).

10.1128/mSystems.00741-19.4 TABLE S3 Mean relative abundance of the predicted functional groups of archaea (%). Download Table S3, XLSX file, 0.01 MB. Copyright © 2020 Wang et al. 2020 Wang et al. This content is distributed under the terms of the Creative Commons Attribution 4.0 International license.

10.1128/mSystems.00741-19.5 TABLE S4 Mean relative abundance of the predicted functional groups of bacteria (%). Download Table S4, XLSX file, 0.01 MB. Copyright © 2020 Wang et al. 2020 Wang et al. This content is distributed under the terms of the Creative Commons Attribution 4.0 International license.

10.1128/mSystems.00741-19.6 TABLE S5 Mean relative abundance of the predicted functional groups of fungi (%). Download Table S5, XLSX file, 0.01 MB. Copyright © 2020 Wang et al. 2020 Wang et al. This content is distributed under the terms of the Creative Commons Attribution 4.0 International license.

### Relationships between microbial communities and soil properties.

**FIG 6:**
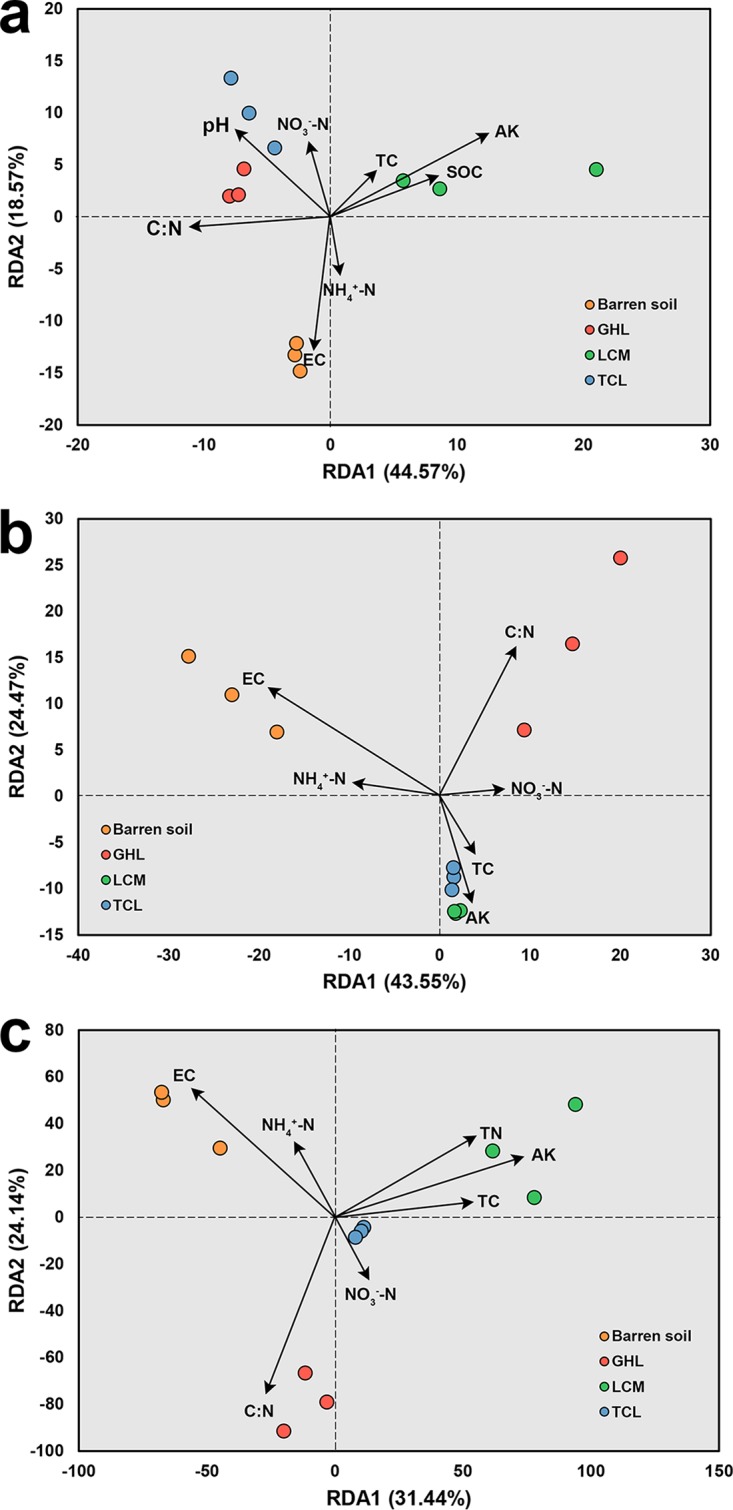
RDA plot depicting the correlation between soil properties and archaeal (a), bacterial (b), and fungal (c) communities in distal-rhizosphere soil.

Redundancy analysis (RDA) was applied to illustrate the correlations between soil properties and microbial community composition in the barren soil and the phytoremediated soils (represented by the distal-rhizosphere soils) (Fig. 6). The first two RDA axes explained 63.14%, 68.02% and 55.58% of the total variation in the archaeal, bacterial, and fungal community structures, respectively. The RDA results showed that soil EC had the highest correlation with the separation of microbial communities between the barren soil and the phytoremediated soils. A number of soil physicochemical properties were strongly correlated with the microbial community composition in the three phytoremediated soils (Fig. 6).

## DISCUSSION

Phytoremediation has several advantages over other remediation techniques for salt-affected soil, such as its relatively low cost compared with chemical remediation (39) and high efficiency in preventing salt leaching into the ground water due to its accumulation in plant shoots (40). However, many aspects of this process, such as the role of microbes and the performance of specific plant species, are still not clear (10). In this study, we found that the introduction of three salt-tolerant plant species, G. hirsutum Linn., T. chinensis Lour., and L. chinense Mill., effectively decreased soil EC and increased soil nutrient contents. TCL showed the highest efficiency in decreasing soil salinity, while the largest increase in soil carbon and nitrogen was achieved in the LCM treatment. Simultaneously, the three salt-tolerant plant species altered the soil microbial communities and displayed species-specific effects on the archaeal, bacterial, and fungal communities. To the best of our knowledge, this is the first study that has evaluated the impact of phytoremediation across plant species in a Chinese coastal saline soil in terms of both soil physicochemical and microbial properties.

Microbial diversity is a pivotal characteristic of soil ecosystems, and it has already been used as an indicator of soil quality (41). Our results showed that the microbial diversity changed differently under the remediation conditions with the three salt-tolerant plant species. Archaeal richness was higher in the barren soil than in the phytoremediated soils, and this may be associated with the extinction of halophiles, since a number of halophiles were identified as the dominant indicator taxa in the archaeal communities in the barren soil but not in the remediated soils (Fig. 3). However, bacterial and fungal richness was higher in the remediated soils than in the barren soil. This is consistent with previous studies demonstrating elevated bacterial diversity in saline soils remediated by Atriplex triangularis and Suaeda glauca (11). The flourishing of bacterial and fungal communities in the phytoremediated soils could be tentatively attributed to the decrease in soil salinity and the increase in soil carbon and nutrients (Table 1). The high salt concentrations in saline soil impose great restrictions on microbial colonization and growth. The elimination of this limiting factor could result in a more open system that would accept more taxa that show low resistance to high salinity.

Plant species-specific effects on microbial communities can be attributed to the composition of root exudates (42). In this study, a stronger influence of plant species was observed on the fungal communities than on the archaeal and bacterial communities (Fig. 1b to d). This could be because fungi are heterotrophs and, compared with bacteria and archaea, are more dependent on the carbon source provided by plants. The profound effects of organic resources on the fungal community have also been demonstrated in agricultural (43, 44) and forest (45) soils.

Previous studies have illustrated the key role of salinity in determining the soil microbial community composition, not only in salt-affected habitats (46) but also through meta-analyses performed with samples from diverse, globally distributed natural environments (47). Consistent with these findings, we also found that EC was the most important environmental factor causing the separation of the microbial communities between the barren soil and the phytoremediated soils (represented by the distal-rhizosphere soils) (Fig. 6). In the treatment with the largest reduction in soil salinity (TCL), the highest diversity of bacteria and fungi was discovered in the phytoremediated soils (Fig. 4). However, the levels of SOC, TC, TN, AK, AP, NH_4_^+^-N and NO_3_^−^-N in the distal rhizosphere were higher in LCM than in TCL (Table 1). One explanation for this result could be that salinity plays a more important role than soil carbon and nutrients in determining the microbial diversity in the investigated soil. Meanwhile, it should be noted that the bacterial and fungal community structure could be strongly influenced by the composition of the root exudates (26, 48). The larger amount of soil carbon achieved in LCM might not support a more diverse microbial community than that in TCL. Therefore, future research is needed to investigate the composition of soil carbon in the different treatments.

The functional prediction for the bacterial OTUs revealed that aerobic chemoheterotrophy was a dominant function in the barren soil and that its abundance increased in the distal rhizosphere, proximal rhizosphere, and rhizoplane of all three phytoremediation treatments (see Table S4 in the supplemental material). In a previous study, this function was also found to be dominant in sediment samples from the Bohai Sea (49), which is near our sampling site, suggesting the prevalence of this function in this area. One possible explanation for the increased abundance of this function in the remediated soils is that the carbon released from the plants enhanced the growth of the chemoheterotrophic microorganisms. It should be noted that the classification of the OTUs into functional groups was performed based on the current literature; this method has several limitations, such as the possible false generalization of a function from cultured members to all members of a taxon, as mentioned previously (50).

### Conclusion.

In a successfully established coastal saline soil remediation experiment, different remediation efficacies were achieved among different salt-tolerant plant species. The composition of the bacterial, archaeal, and fungal communities in all the rhizocompartments was significantly different from that in the barren soil, suggesting that salt-tolerant plants greatly altered the soil microbial communities. The fungal community clearly varied across plant species. The key taxa that respond to remediation of each plant were identified and could be potentially used as bioindicators for phytoremediation. Soil EC was identified to be crucial for shaping the microbial community in the investigated coastal saline soil. This study improved our understanding of the potential roles of host-associated microbial communities in microbe-mediated plant salt tolerance in costal saline soil. In particular, fungi may be more important than bacteria and archaea for distinguishing among root-zone microbiomes of different salt-tolerant plant species.

## MATERIALS AND METHODS

### Site description and soil sampling.

The field experiment was set up in a research zone of the Chinese Academy of Sciences for efficient utilization of coastal saline soils in Haixing County, Hebei Province, China (117°33ʹ49ʹʹE, 38°10ʹ02ʹʹN). The annual average temperature and precipitation are 12.1°C and 582.3 mm, respectively. A homogeneous coastal area was chosen to ensure that all the experimental plots had uniform soil properties. The field experiment was established in 2005 with four treatments: (i) barren land (no vegetation) and three treatments planted with (ii) Gossypium hirsutum Linn., (iii) Tamarix chinensis Lour., and (iv) Lycium chinense Mill. Each treatment had three replicate plots of 10 m by 10 m.

Samples from the barren land and the four rhizocompartments of the three salt-tolerant species were collected on 24 October 2017. In this study, we defined the soil surrounding the plant roots as the proximal-rhizosphere soil and the soil in an experimental plot without visible roots as the distal-rhizosphere soil (see Fig. S1 in the supplemental material). For each soil sample, five soil cores in each plot were randomly collected to form a composite sample. The barren soil, distal-rhizosphere, and proximal-rhizosphere soils were sieved through 2-mm-pore-size mesh screens to remove impurities. The soils collected for DNA extraction and chemical analysis were stored at −80°C and 4°C, respectively. Rhizoplane and endosphere samples were collected according to the methods described in previous studies (51, 52) with some minor modifications. Briefly, after three rinses with sterile water were performed to completely remove the soils on the surface, the roots were placed in a 50-ml Falcon tube with 30 ml phosphate-buffered saline (PBS) and sonicated for 1 min at 40 kHz in an ultrasonic cleaner (Jiemei-KS-5200DE; Kunshan, China). Then, the roots were removed, and the solutions were centrifuged for 2 min at 10,000 × *g*. The precipitates were collected as rhizoplane samples and stored at −80°C. The sonicated roots were washed with sterile water three times, soaked for 5 min in 30 ml 2% sodium hypochlorite, and then cleaned again with sterile water. PCRs using the washed waste as the template were performed to check whether all the microbes on the root surface had been removed. The roots were stored at −80°C until DNA extraction.

10.1128/mSystems.00741-19.1 FIG S1 Schematic diagram of the sampling locations. Download FIG S1, DOCX file, 0.5 MB. Copyright © 2020 Wang et al. 2020 Wang et al. This content is distributed under the terms of the Creative Commons Attribution 4.0 International license.

### DNA extraction.

The total genomic DNA from the samples was extracted using a FastDNA spin kit for soil (MP Biomedicals, Santa Ana, CA, USA) following the manufacturer’s protocol. For the endosphere samples, liquid nitrogen was added, and the samples were ground before total genomic DNA extraction was performed. The extracted DNA was purified using a DNeasy PowerClean cleanup kit (MoBio Laboratories, Carlsbad, CA, USA). The concentration and purity of the extracted DNA were examined on a NanoDrop One system (NanoDrop Technologies, Wilmington, DE, USA) and stored at –20°C until molecular analysis.

### Soil physical and chemical analyses.

The soil physical and chemical characteristics were measured according to a procedure described previously by Chu and Grogan (53). The soil water content (WC) was measured by oven drying of the soil for 6 h at 105°C. The soil pH was determined in a 1:5 (wt/vol) mixture of soil and water using a pH meter (Mettler-Toledo FE28, Switzerland). The soil samples were ground and then sieved through 150-μm-pore-size mesh screens to measure the total carbon (TC) and total nitrogen (TN) concentrations using a CHNOS elemental analyzer (Vario MAX, Elementar, Germany). Ammonia nitrogen (NH4^+^-N) and nitrate nitrogen (NO_3_^−^-N) were extracted with 2 M KCl solution. The NO_3_^−^-N level was determined by a dual-wavelength scheme (54) using an UV spectrophotometer (UV-6100S; Metash). The NH_4_^+^-N level was determined using the indophenol blue spectrophotometer method (625-nm wavelength). The soil organic carbon (SOC) content was measured using the K_2_Cr_2_O_7_-H_2_SO_4_ oxidation method (55). The soil-available potassium (AK) and available phosphorus (AP) were extracted by using 1 M ammonium acetate and 0.5 M NaHCO_3_, respectively. The AK was measured using a flame photometer (FP640; INASA Scientific Instrument Co., Ltd., China), and the AP was measured using the molybdenum blue method (56). The electrical conductivity (EC) of the soil samples was measured in 1:5 (wt/vol) mixture of moist soil and boiled water using a conductivity meter (DDS-307A; INASA Scientific Instrument Co., Ltd., China).

### PCR amplification and sequencing.

For archaea and bacteria, the V4 region of the 16S rRNA gene was amplified using the primer pair 515F (5′-GTGYCAGCMGCCGCGGTAA-3′) and 806R (5′-GGACTACNVGGGTWTCTAAT-3′) (57). For fungi, the ITS1 region of the fungal internal transcribed spacer (ITS) was amplified with the primer pair ITS1f (5′-CTTGGTCATTTAGAGGAAGTAA-3′) and ITS2 (5′-GCTGCGTTCTTCATCGATGC-3′) (57). PCR amplifications were conducted in a 25-μl system containing 12.5 μl 2× premix *Ex Taq* (TaKaRa), 0.5 μl of each primer (10 μM), 1 μl (20 ng μl^−1^) template DNA, and 10.5 μl sterile double-distilled water. The thermal cycling profile for PCR was as follows: an initial denaturation at 95°C for 10 min; 28 cycles of 30 s at 95°C and 1 min at 50°C (for bacteria) or 60°C (for fungi) for annealing; 1 min at 72°C for extension; with a final extension for 10 min at 72°C. The PCR products were checked by electrophoresis on a 1.5% agarose gel and were then purified with AMPure XP beads (Beckman Coulter Inc., Brea, CA, USA) following the manufacturer’s protocol. A subsequent eight-cycle PCR was performed to add the Illumina sequencing adapters and dual-index barcodes for each amplicon. The PCR products were purified with AMPure beads (Beckman Coulter Inc., Brea, CA, USA) and sequenced on an Illumina MiSeq platform.

### Analysis of the high-throughput sequencing data.

The bioinformatic analyses were performed mainly with QIIME (Quantitative Insights Into Microbial Ecology, version 1.9.1) (58) according to the methods described in a previous study (59). In brief, the barcode sequences and adapter sequences and 55 bases of low quality at the end of the reads were discarded. Then, the paired reads were merged using the fastq-join algorithm (60). The minimum overlap length was set to 20 bp, and a maximum of 10% mismatches were allowed within the overlap region. Low-quality sequences (lengths of <200 bp or Phred quality scores of <20) and chimeras of microbial sequences were removed using the USEARCH package (61) with the UCHIME algorithm (62). The clean data were then clustered into operational taxonomic units (OTUs) at 97% similarity using the UCLUST method (61). The most abundant sequence within each OTU was selected as the representative sequence. Taxonomy was assigned using the RDP Classifier (63) against the Silva database (version 132) (64) for archaeal and bacterial OTUs and the UNITE database (QIIME released, version 7.0) (65) for fungal OTUs. The OTUs that were not assigned to bacteria, archaea, or fungi and the bacterial OTUs assigned to chloroplasts or mitochondria and singleton OTUs were removed prior to further analysis. The OTU tables corresponding to archaea, bacteria, and fungi were subsampled to 700, 11,000, and 20,000 sequences per sample, respectively, for statistical analysis. The functional annotation of the archaeal and bacterial taxa in the different treatments was performed using the Functional Annotation of Prokaryotic Taxa (FAPROTAX) database (50). The functional profiles of the fungi in the different treatments were annotated using FUNGuild (version 1.0) (66). Only the OTUs that were assigned a trophic mode with a confidence ranking of “highly probable” or “probable” in FUNGuild were kept for further analysis.

### Statistical analysis.

Chao1 index (67) was calculated to compare the level of diversity of the microbial communities in the different treatments. Nonmetric multiple-dimensional scaling (NMDS) based on Bray-Curtis distances was performed to show the differences in community composition among treatments. Redundancy analysis (RDA) was performed to test for the relationships between soil characteristics and microbial community composition using the vegan package in R (version 3.4.3) (68). Dufrene-Legendre indicator species analysis (69) was performed to identify indicator species in the barren soil and phytoremediated soils. The significance of the differences in soil properties between treatments was determined by Duncan’s test at a 95% confidence level in SPSS for Windows (version 20.0; IBM Corporation, Armonk, NY, USA). Venn diagrams showing the unique and shared OTUs of the three phytoremediation treatments were drawn in R using the package gplots (70).

### Data availability.

All sequencing data used in this study are available in the European Nucleotide Archive under the accession number PRJEB34423.
