# Supplementary material for: Long-Term Phytoremediation of Coastal Saline Soil Reveals Plant Species-Specific Patterns of Microbial Community Recruitment
Source: mSystems. 2020 Mar 3;5(2):e00741-19. doi: 10.1128/mSystems.00741-19 (PMC7055657; doi:10.1128/mSystems.00741-19)
Supplement: TABLE S1 [file mSystems.00741-19-st001.docx]

**TABLE** **S1** Dominant bacterial OTUs enriched in the rhizoplane and/or endosphere^a^.

| Treatment | OTU | Distal-rhizosphere | Proximal-rhizosphere | Rhizoplane | Endosphere | Taxonomy |
| --- | --- | --- | --- | --- | --- | --- |
| GHL | OTU146527 | 0.01 | 0.03 | 4.53 | 2.37 | g_*Ilumatobacter* |
|  | OTU32941 | 0.00 | 0.01 | 1.37 | 1.26 | g_*Ilumatobacter* |
|  | OTU203257 | 0.06 | 0.08 | 0.77 | 13.33 | f_ *Rhizobiaceae* |
|  | OTU18796 | 0.01 | 0.04 | 0.59 | 2.27 | f_*Sphingomonadaceae* |
|  | OTU76386 | 0.03 | 0.09 | 0.38 | 2.18 | g_*Novosphingobium* |
|  | OTU114111 | 0.01 | 0.02 | 0.39 | 2.23 | g_*Novosphingobium* |
|  | OTU208404 | 0.06 | 0.08 | 0.96 | 3.10 | g_*Sphingobium* |
|  | OTU157366 | 0.01 | 0.06 | 0.03 | 2.78 | f_*Enterobacteriaceae* |
|  | OTU33196 | 0.00 | 0.00 | 0.01 | 1.39 | g_*Pantoea* |
| LCM | OTU59709 | 0.07 | 0.05 | 0.46 | 5.91 | g_*Mycobacterium* |
|  | OTU64064 | 0.02 | 0.05 | 4.52 | 0.05 | g_*Glutamicibacter* |
|  | OTU7192 | 0.02 | 0.03 | 3.22 | 0.35 | f_*Nitrosococcaceae* |
|  | OTU149372 | 0.00 | 0.00 | 0.03 | 5.11 | g_*Amycolatopsis* |
|  | OTU200505 | 0.01 | 0.03 | 0.15 | 11.87 | g_*Lechevalieria* |
|  | OTU57481 | 0.05 | 0.04 | 1.25 | 0.73 | g_*Streptomyces* |
|  | OTU103703 | 0.03 | 0.02 | 0.73 | 12.34 | g_*Streptomyces* |
|  | OTU24429 | 0.02 | 0.02 | 0.63 | 1.06 | g_*Solirubrobacter* |
|  | OTU46251 | 0.00 | 0.01 | 1.53 | 0.01 | g_*Bacillus* |
|  | OTU96242 | 0.02 | 0.02 | 3.71 | 0.08 | g_*Bacillus* |
|  | OTU168498 | 0.00 | 0.00 | 0.96 | 0.01 | g_*Solibacillus* |
|  | OTU184242 | 0.00 | 0.00 | 0.08 | 2.95 | g_*Bosea* |
|  | OTU150858 | 0.00 | 0.00 | 2.09 | 0.37 | f_*Rhizobiaceae* |
|  | OTU203257 | 0.04 | 0.02 | 1.25 | 0.56 | f_ *Rhizobiaceae* |
|  | OTU218791 | 0.01 | 0.02 | 0.92 | 1.31 | g_*Achromobacter* |
|  | OTU157366 | 0.03 | 0.02 | 5.06 | 0.05 | f_*Enterobacteriaceae* |
|  | OTU33196 | 0.00 | 0.00 | 2.78 | 0.02 | g_*Pantoea* |
|  | OTU67359 | 0.05 | 0.01 | 1.39 | 0.02 | g_*Acinetobacter* |
|  | OTU83647 | 0.05 | 0.01 | 4.81 | 0.19 | g_*Acinetobacter* |
|  | OTU76106 | 0.41 | 0.41 | 2.97 | 0.10 | g_*Pseudomonas* |
| TCL | OTU147871 | 0.58 | 0.34 | 1.46 | 0.27 | o_*Actinomarinales* |
|  | OTU206485 | 0.00 | 0.00 | 0.16 | 1.84 | f_*Microtrichaceae* |
|  | OTU4077 | 0.00 | 0.00 | 0.11 | 6.15 | o_*Microtrichales* |
|  | OTU211927 | 0.01 | 0.01 | 0.15 | 9.80 | f_*Kineosporiaceae* |
|  | OTU57481 | 0.03 | 0.03 | 0.41 | 2.71 | g_*Streptomyces* |
|  | OTU193730 | 0.01 | 0.02 | 0.18 | 1.42 | g_*Rhodomicrobium* |
|  | OTU210650 | 0.00 | 0.00 | 0.08 | 1.50 | g_*Rhodomicrobium* |
|  | OTU199955 | 0.57 | 0.76 | 1.03 | 0.03 | f_*Desulfuromonadaceae* |
|  | OTU174417 | 0.00 | 0.00 | 0.00 | 3.10 | g_*Candidatus* *Phytoplasma* |

^a^The bacterial OTUs listed in this table were significantly enriched (P < 0.05, Kruskal-Wallis test) in the rhizoplane and/or endosphere compared with the distal/proximal rhizosphere and had a relative abundance >1%.
