# Supplementary material for: Long-Term Phytoremediation of Coastal Saline Soil Reveals Plant Species-Specific Patterns of Microbial Community Recruitment
Source: mSystems. 2020 Mar 3;5(2):e00741-19. doi: 10.1128/mSystems.00741-19 (PMC7055657; doi:10.1128/mSystems.00741-19)
Supplement: TABLE S2 [file mSystems.00741-19-st002.docx]

**TABLE S2** Dominant fungal OTUs enriched in the rhizoplane and/or endosphere^a^.

| Treatment | OTU | Distal-rhizosphere | Proximal-rhizosphere | Rhizoplane | Endosphere | Taxonomy |
| --- | --- | --- | --- | --- | --- | --- |
| GHL | OTU3702 | 0.11 | 0.02 | 1.59 | 0.27 | g_*Aspergillus* |
|  | OTU1300 | 11.41 | 0.72 | 37.96 | 2.40 | g_*Talaromyces* |
|  | OTU909 | 0.00 | 0.00 | 0.00 | 1.48 | k_Fungi |
|  | OTU3616 | 0.00 | 0.00 | 0.00 | 2.70 | k_Fungi |
|  | OTU7279 | 0.00 | 0.00 | 0.00 | 1.03 | k_Fungi |
|  | OTU8691 | 0.00 | 0.00 | 0.00 | 1.24 | k_Fungi |
|  | OTU10780 | 0.00 | 0.00 | 0.00 | 1.15 | k_Fungi |
| LCM | OTU3991 | 1.1 | 1.04 | 2.50 | 0.00 | g_*Arthrographis* |
|  | OTU9306 | 0.25 | 1.01 | 0.17 | 8.24 | g_*Byssothecium* |
|  | OTU599 | 1.44 | 2.28 | 9.12 | 0.42 | g_*Gibellulopsis* |
|  | OTU1860 | 0.15 | 0.07 | 0.76 | 24.9 | g_*Purpureocillium* |
|  | OTU9077 | 0.17 | 0.33 | 16.96 | 8.07 | g_*Phialemoniopsis* |
| TCL | OTU6589 | 0.05 | 0.00 | 0.02 | 1.70 | p_Ascomycota |
|  | OTU8338 | 0.37 | 0.41 | 0.86 | 1.09 | g_*Cladosporium* |
|  | OTU2063 | 0.43 | 0.28 | 2.40 | 2.46 | g_*Alternaria* |
|  | OTU1913 | 0.16 | 0.35 | 0.95 | 20.7 | g_*Exophiala* |
|  | OTU5608 | 0.27 | 0.28 | 2.56 | 0.00 | g_*Penicillium* |
|  | OTU1300 | 0.12 | 2.54 | 6.03 | 0.01 | g_*Talaromyces* |
|  | OTU2263 | 0.67 | 0.63 | 1.45 | 1.38 | g_*Plectosphaerella* |
|  | OTU2539 | 0.01 | 0.08 | 0.40 | 15.69 | o_*Hypocreales* |
|  | OTU8583 | 0.46 | 0.11 | 1.39 | 0.75 | g_*Fusarium* |
|  | OTU1860 | 0.14 | 0.66 | 4.70 | 0.22 | g_*Purpureocillium* |
|  | OTU10478 | 0.89 | 0.12 | 0.13 | 4.52 | g_*Halosarpheia* |
|  | OTU10712 | 0.15 | 0.18 | 1.89 | 0.00 | g_*Staphylotrichum* |
|  | OTU2689 | 0.01 | 0.26 | 1.51 | 0.00 | k_Fungi |
|  | OTU9467 | 0.00 | 0.00 | 0.00 | 1.83 | k_Fungi |
|  | OTU10259 | 0.00 | 0.00 | 0.02 | 1.41 | k_Fungi |

^a^The fungal OTUs listed in this table were significantly enriched (P < 0.05, Kruskal-Wallis test) in the rhizoplane and/or endosphere compared with the distal/proximal rhizosphere and had a relative abundance >1%.
