# Supplementary figures and images for: Long-Term Phytoremediation of Coastal Saline Soil Reveals Plant Species-Specific Patterns of Microbial Community Recruitment
Source: mSystems. 2020 Mar 3;5(2):e00741-19. doi: 10.1128/mSystems.00741-19 (PMC7055657; doi:10.1128/mSystems.00741-19)

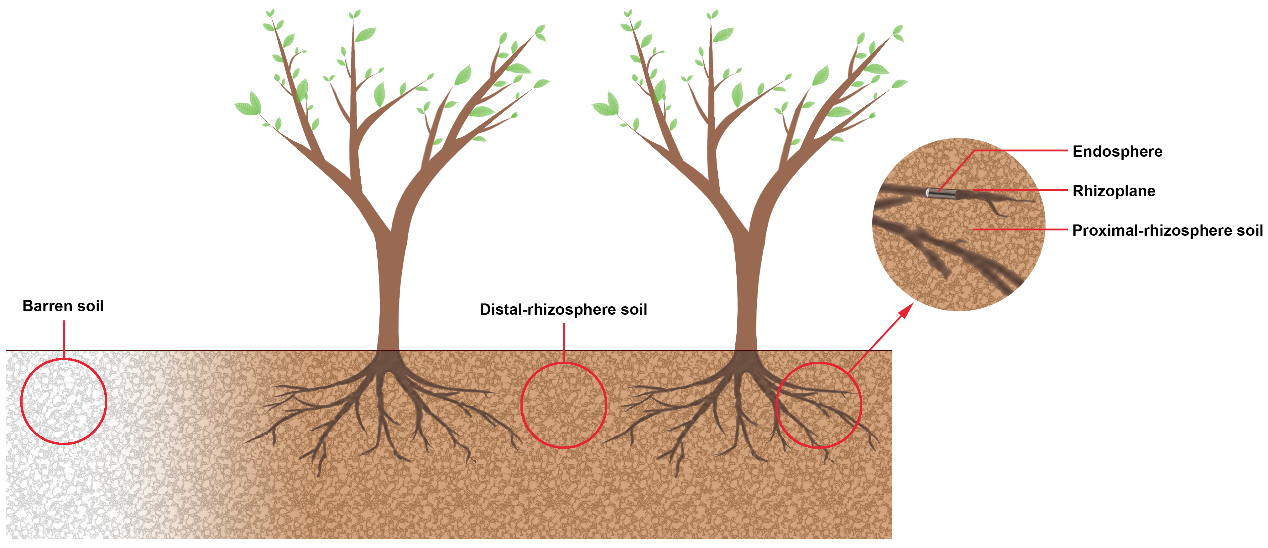

Supplement: FIG S1 [file mSystems.00741-19-sf001.docx]
